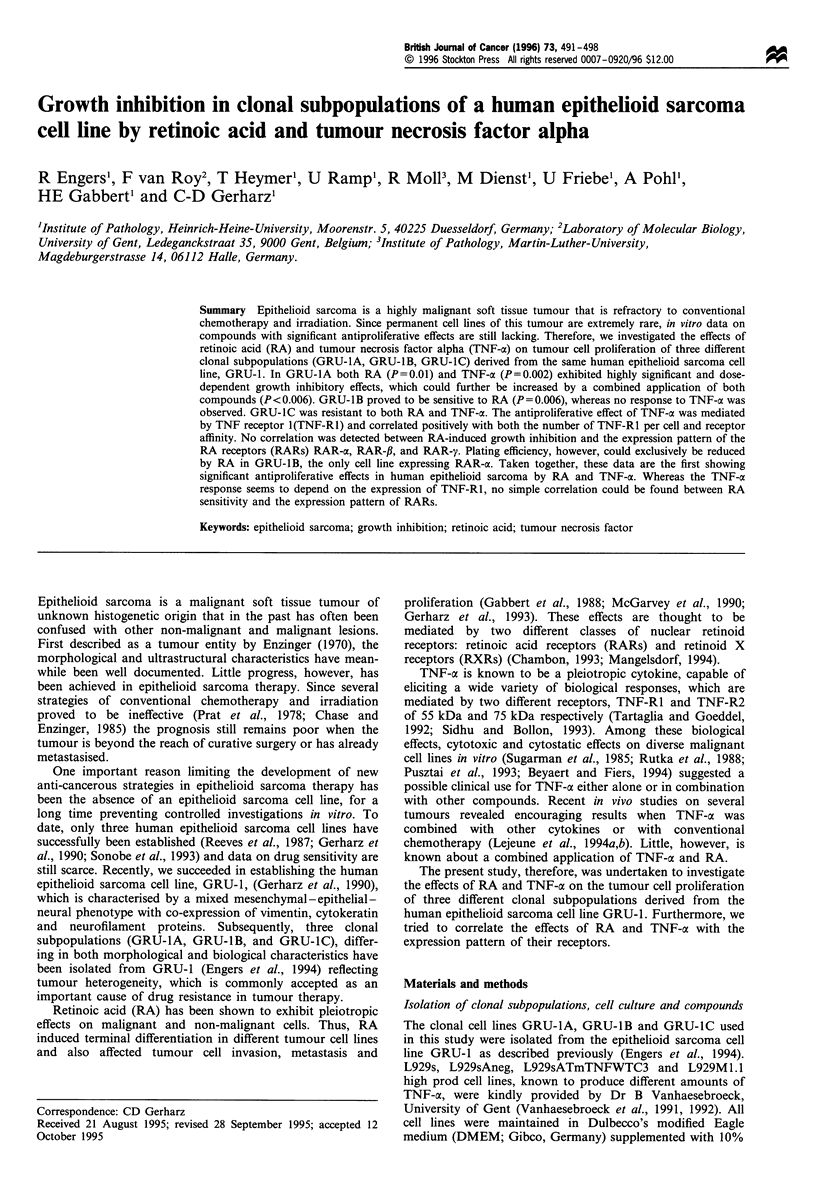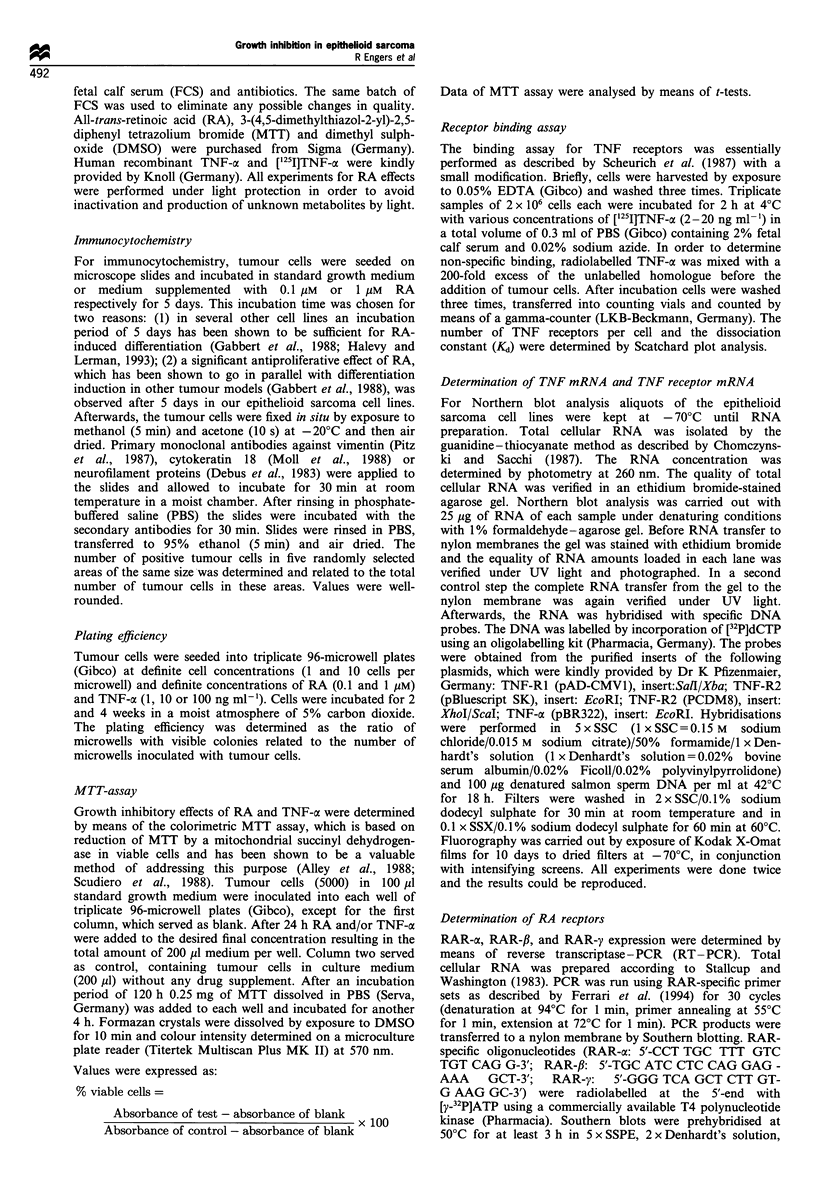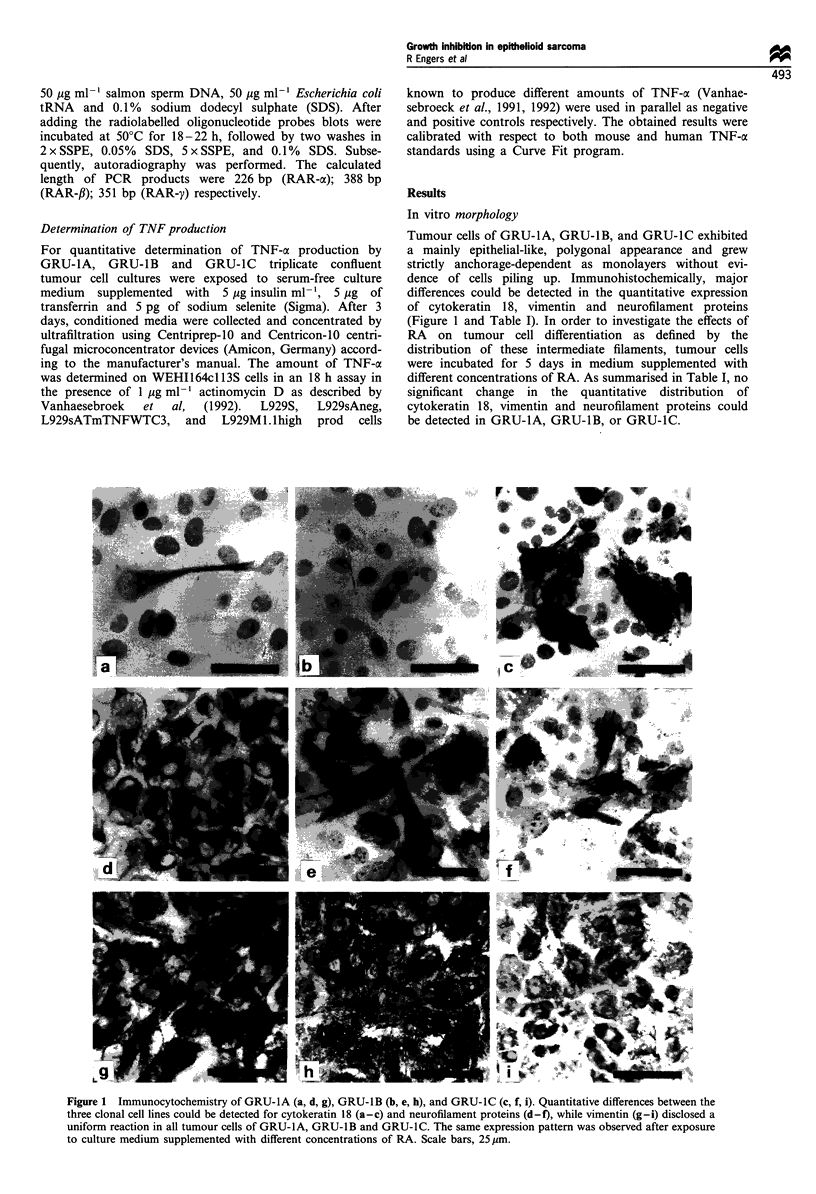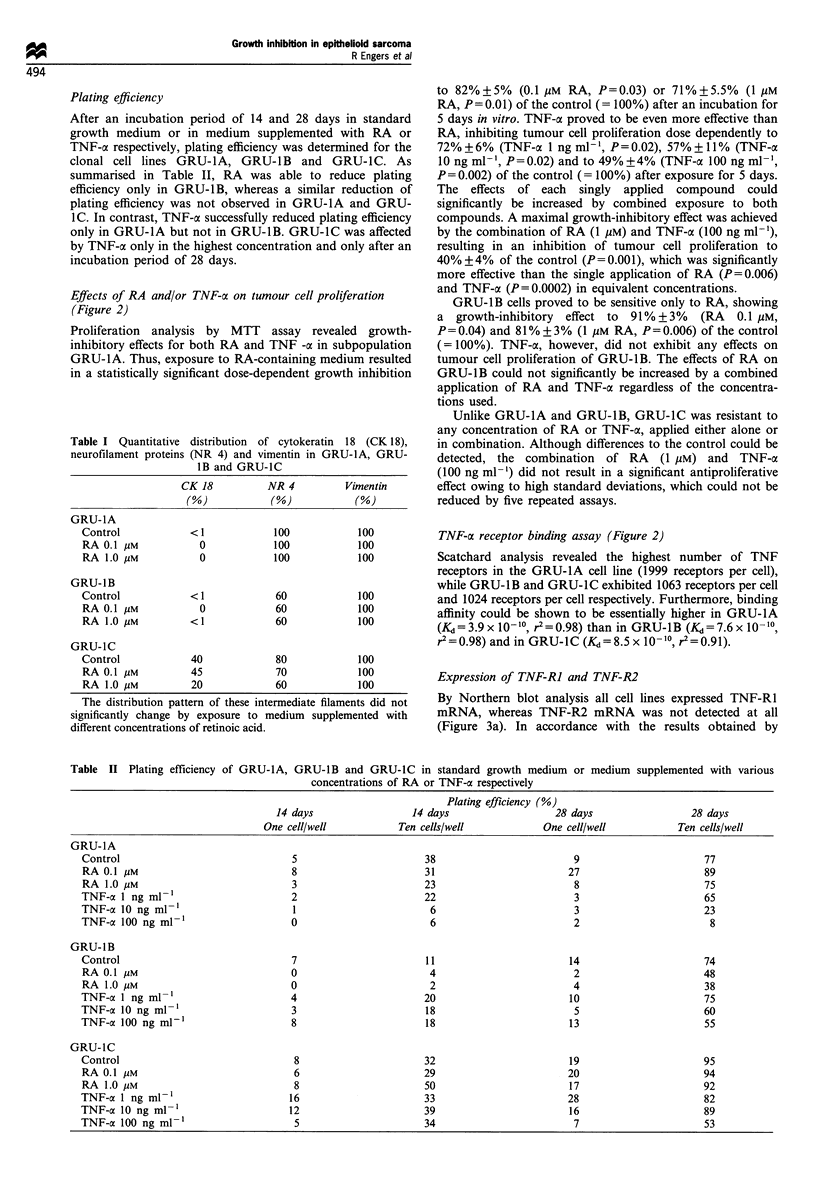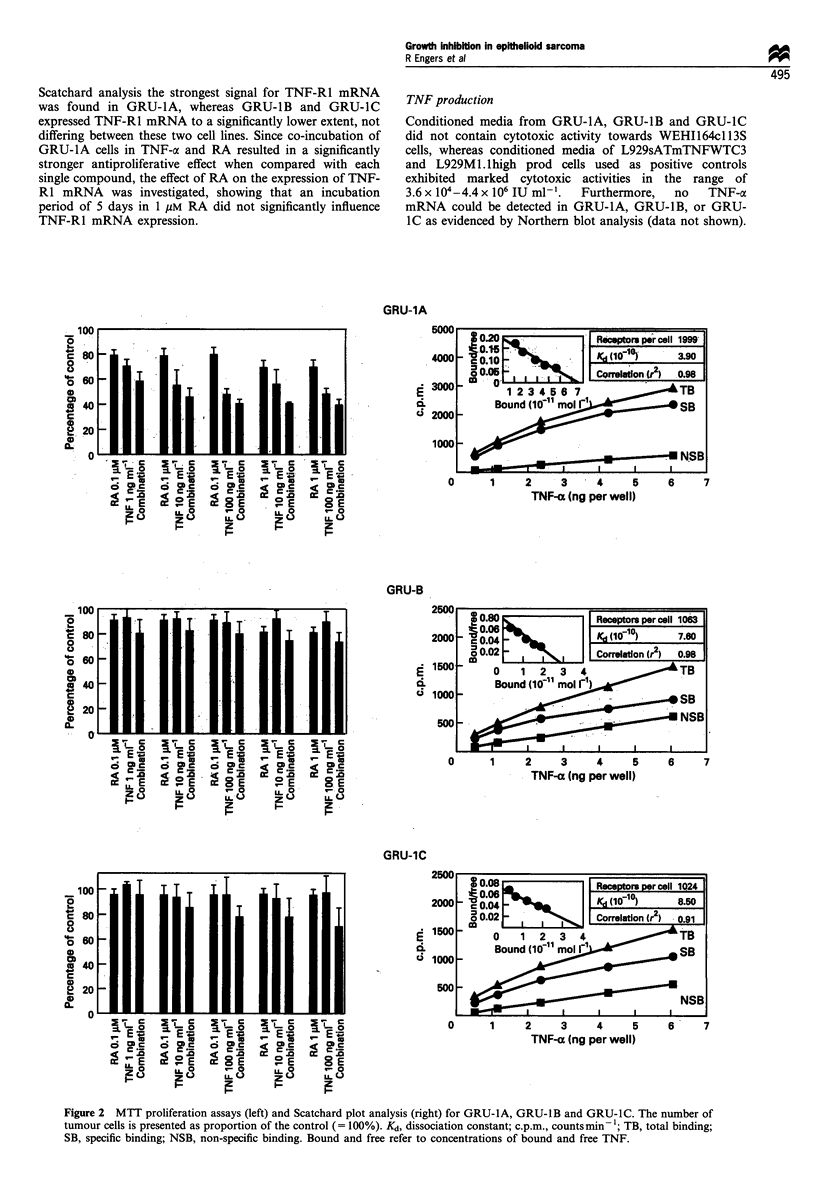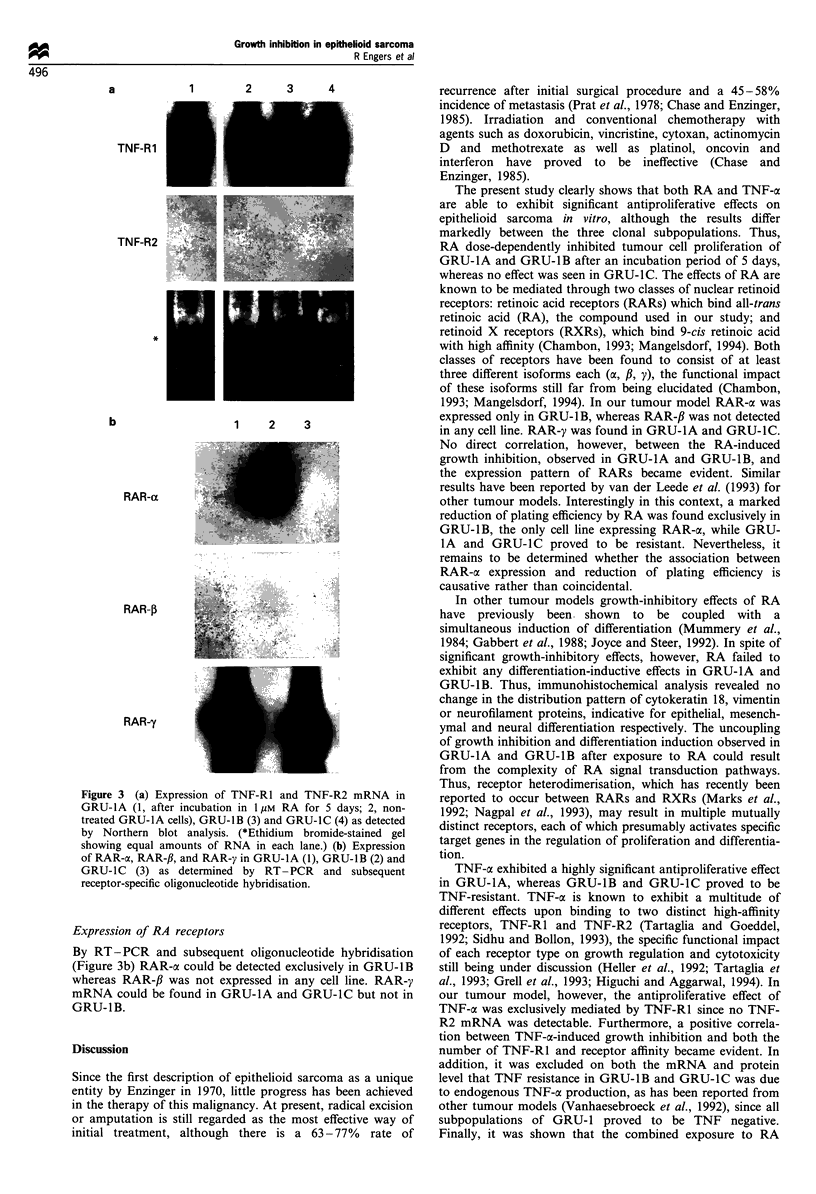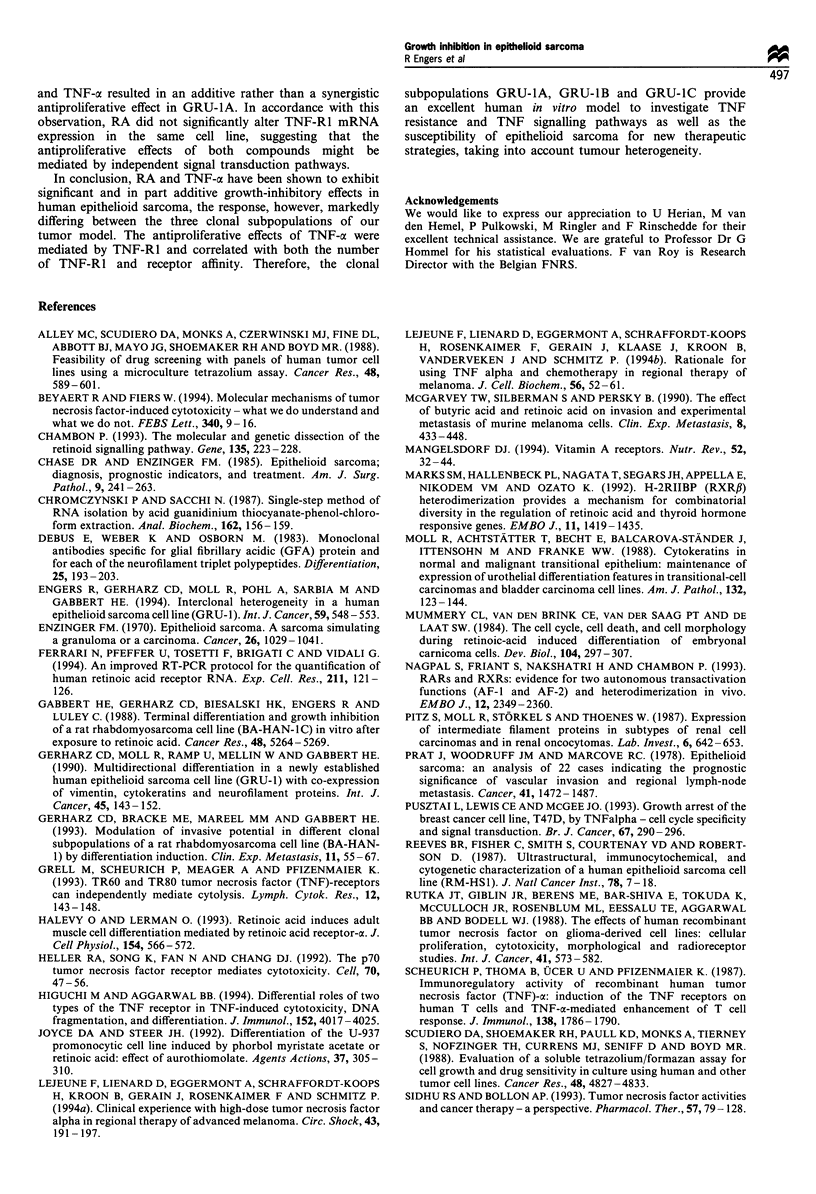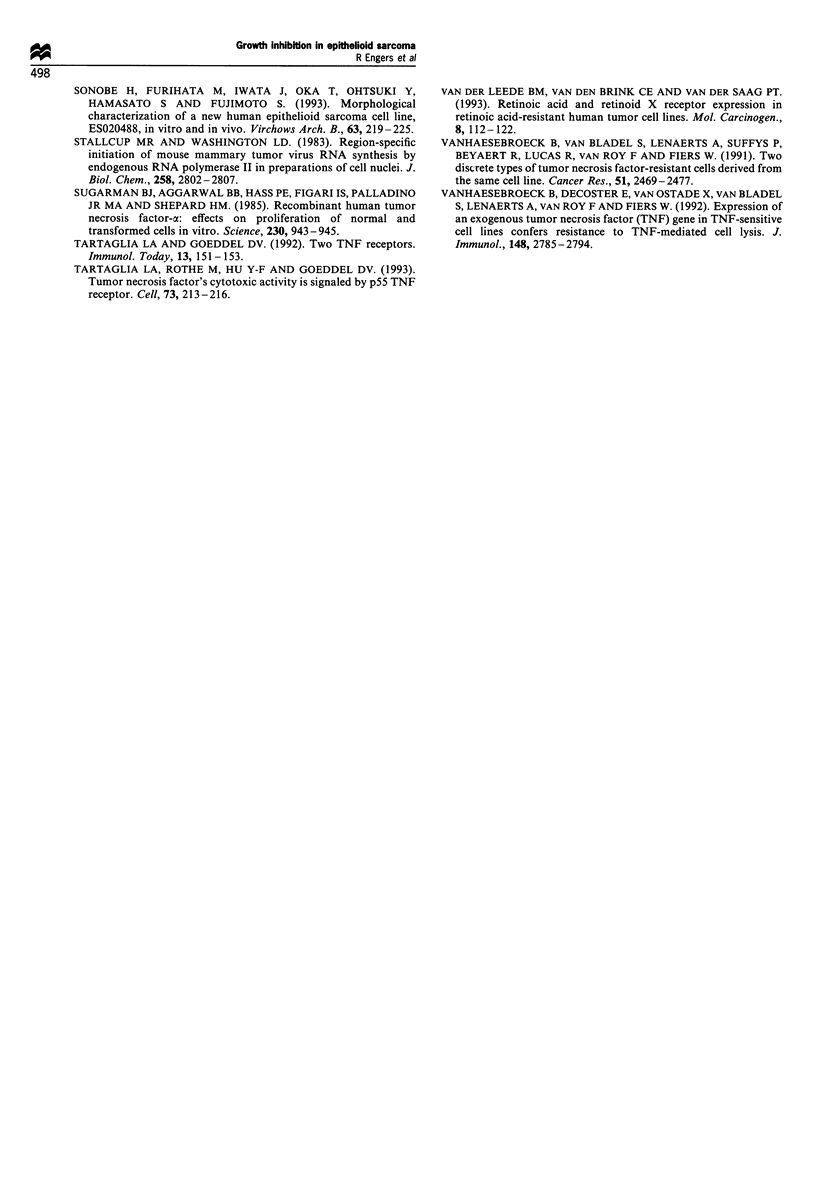# Growth inhibition in clonal subpopulations of a human epithelioid sarcoma cell line by retinoic acid and tumour necrosis factor alpha.

**DOI:** 10.1038/bjc.1996.86

**Published:** 1996-02

**Authors:** R. Engers, F. van Roy, T. Heymer, U. Ramp, R. Moll, M. Dienst, U. Friebe, A. Pohl, H. E. Gabbert, C. D. Gerharz

**Affiliations:** Institute of Pathology, Heinrich-Heine University, Dusseldorf, Germany.

## Abstract

**Images:**


British Journal of Cancer (1996) 73, 491-498
© 1996 Stockton Press All rights reserved 0007-0920/96 $12.00

### Growth inhibition in clonal subpopulations of a human epithelioid sarcoma cell line by retinoic acid and tumour necrosis factor alpha

R Engers[1], F van Roy[2], T Heymer[1], U Ramp[1], R Moll[3], M Dienst[1], U Friebe[1], A Pohl[1], HE Gabbert[1] and C-D Gerharz[1]

[1]*Institute of Pathology, Heinrich-Heine-University, Moorenstr. 5, 40225 Duesseldorf, Germany;* [2]*Laboratory of Molecular Biology, University of Gent, Ledeganckstraat 35, 9000 Gent, Belgium;* [3]*Institute of Pathology, Martin-Luther-University, Magdeburgerstrasse 14, 06112 Halle, Germany.*

**Summary** Epithelioid sarcoma is a highly malignant soft tissue tumour that is refractory to conventional chemotherapy and irradiation. Since permanent cell lines of this tumour are extremely rare, *in vitro* data on compounds with significant antiproliferative effects are still lacking. Therefore, we investigated the effects of retinoic acid (RA) and tumour necrosis factor alpha (TNF-α) on tumour cell proliferation of three different clonal subpopulations (GRU-1A, GRU-1B, GRU-1C) derived from the same human epithelioid sarcoma cell line, GRU-1. In GRU-1A both RA ($P=0.01$) and TNF-α ($P=0.002$) exhibited highly significant and dose-dependent growth inhibitory effects, which could further be increased by a combined application of both compounds ($P<0.006$). GRU-1B proved to be sensitive to RA ($P=0.006$), whereas no response to TNF-α was observed. GRU-1C was resistant to both RA and TNF-α. The antiproliferative effect of TNF-α was mediated by TNF receptor 1(TNF-R1) and correlated positively with both the number of TNF-R1 per cell and receptor affinity. No correlation was detected between RA-induced growth inhibition and the expression pattern of the RA receptors (RARs) RAR-α, RAR-β, and RAR-γ. Plating efficiency, however, could exclusively be reduced by RA in GRU-1B, the only cell line expressing RAR-α. Taken together, these data are the first showing significant antiproliferative effects in human epithelioid sarcoma by RA and TNF-α. Whereas the TNF-α response seems to depend on the expression of TNF-R1, no simple correlation could be found between RA sensitivity and the expression pattern of RARs.

**Keywords:** epithelioid sarcoma; growth inhibition; retinoic acid; tumour necrosis factor

Epithelioid sarcoma is a malignant soft tissue tumour of unknown histogenetic origin that in the past has often been confused with other non-malignant and malignant lesions. First described as a tumour entity by Enzinger (1970), the morphological and ultrastructural characteristics have meanwhile been well documented. Little progress, however, has been achieved in epithelioid sarcoma therapy. Since several strategies of conventional chemotherapy and irradiation proved to be ineffective (Prat *et al.*, 1978; Chase and Enzinger, 1985) the prognosis still remains poor when the tumour is beyond the reach of curative surgery or has already metastasised.

One important reason limiting the development of new anti-cancerous strategies in epithelioid sarcoma therapy has been the absence of an epithelioid sarcoma cell line, for a long time preventing controlled investigations *in vitro*. To date, only three human epithelioid sarcoma cell lines have successfully been established (Reeves *et al.*, 1987; Gerharz *et al.*, 1990; Sonobe *et al.*, 1993) and data on drug sensitivity are still scarce. Recently, we succeeded in establishing the human epithelioid sarcoma cell line, GRU-1, (Gerharz *et al.*, 1990), which is characterised by a mixed mesenchymal-epithelial-neural phenotype with co-expression of vimentin, cytokeratin and neurofilament proteins. Subsequently, three clonal subpopulations (GRU-1A, GRU-1B, and GRU-1C), differing in both morphological and biological characteristics have been isolated from GRU-1 (Engers *et al.*, 1994) reflecting tumour heterogeneity, which is commonly accepted as an important cause of drug resistance in tumour therapy.

Retinoic acid (RA) has been shown to exhibit pleiotropic effects on malignant and non-malignant cells. Thus, RA induced terminal differentiation in different tumour cell lines and also affected tumour cell invasion, metastasis and proliferation (Gabbert *et al.*, 1988; McGarvey *et al.*, 1990; Gerharz *et al.*, 1993). These effects are thought to be mediated by two different classes of nuclear retinoid receptors: retinoic acid receptors (RARs) and retinoid X receptors (RXRs) (Chambon, 1993; Mangelsdorf, 1994).

TNF-α is known to be a pleiotropic cytokine, capable of eliciting a wide variety of biological responses, which are mediated by two different receptors, TNF-R1 and TNF-R2 of 55 kDa and 75 kDa respectively (Tartaglia and Goeddel, 1992; Sidhu and Bollon, 1993). Among these biological effects, cytotoxic and cytostatic effects on diverse malignant cell lines *in vitro* (Sugarman *et al.*, 1985; Rutka *et al.*, 1988; Pusztai *et al.*, 1993; Beyaert and Fiers, 1994) suggested a possible clinical use for TNF-α either alone or in combination with other compounds. Recent *in vivo* studies on several tumours revealed encouraging results when TNF-α was combined with other cytokines or with conventional chemotherapy (Lejeune *et al.*, 1994*a*,*b*). Little, however, is known about a combined application of TNF-α and RA.

The present study, therefore, was undertaken to investigate the effects of RA and TNF-α on the tumour cell proliferation of three different clonal subpopulations derived from the human epithelioid sarcoma cell line GRU-1. Furthermore, we tried to correlate the effects of RA and TNF-α with the expression pattern of their receptors.

### Materials and methods

### *Isolation of clonal subpopulations, cell culture and compounds*

The clonal cell lines GRU-1A, GRU-1B and GRU-1C used in this study were isolated from the epithelioid sarcoma cell line GRU-1 as described previously (Engers *et al.*, 1994). L929s, L929sAneg, L929sATmTNFWTC3 and L929M1.1 high prod cell lines, known to produce different amounts of TNF-α, were kindly provided by Dr B Vanhaesebroeck, University of Gent (Vanhaesebroeck *et al.*, 1991, 1992). All cell lines were maintained in Dulbecco's modified Eagle medium (DMEM; Gibco, Germany) supplemented with 10%

Correspondence: CD Gerharz
Received 21 August 1995; revised 28 September 1995; accepted 12 October 1995

fetal calf serum (FCS) and antibiotics. The same batch of FCS was used to eliminate any possible changes in quality. All-*trans*-retinoic acid (RA), 3-(4,5-dimethylthiazol-2-yl)-2,5-diphenyl tetrazolium bromide (MTT) and dimethyl sulphoxide (DMSO) were purchased from Sigma (Germany). Human recombinant TNF-α and [$^{125}$I]TNF-α were kindly provided by Knoll (Germany). All experiments for RA effects were performed under light protection in order to avoid inactivation and production of unknown metabolites by light.

*Immunocytochemistry*

For immunocytochemistry, tumour cells were seeded on microscope slides and incubated in standard growth medium or medium supplemented with 0.1 μM or 1 μM RA respectively for 5 days. This incubation time was chosen for two reasons: (1) in several other cell lines an incubation period of 5 days has been shown to be sufficient for RA-induced differentiation (Gabbert *et al.*, 1988; Halevy and Lerman, 1993); (2) a significant antiproliferative effect of RA, which has been shown to go in parallel with differentiation induction in other tumour models (Gabbert *et al.*, 1988), was observed after 5 days in our epithelioid sarcoma cell lines. Afterwards, the tumour cells were fixed *in situ* by exposure to methanol (5 min) and acetone (10 s) at −20°C and then air dried. Primary monoclonal antibodies against vimentin (Pitz *et al.*, 1987), cytokeratin 18 (Moll *et al.*, 1988) or neurofilament proteins (Debus *et al.*, 1983) were applied to the slides and allowed to incubate for 30 min at room temperature in a moist chamber. After rinsing in phosphate-buffered saline (PBS) the slides were incubated with the secondary antibodies for 30 min. Slides were rinsed in PBS, transferred to 95% ethanol (5 min) and air dried. The number of positive tumour cells in five randomly selected areas of the same size was determined and related to the total number of tumour cells in these areas. Values were well-rounded.

*Plating efficiency*

Tumour cells were seeded into triplicate 96-microwell plates (Gibco) at definite cell concentrations (1 and 10 cells per microwell) and definite concentrations of RA (0.1 and 1 μM) and TNF-α (1, 10 or 100 ng ml$^{-1}$). Cells were incubated for 2 and 4 weeks in a moist atmosphere of 5% carbon dioxide. The plating efficiency was determined as the ratio of microwells with visible colonies related to the number of microwells inoculated with tumour cells.

*MTT-assay*

Growth inhibitory effects of RA and TNF-α were determined by means of the colorimetric MTT assay, which is based on reduction of MTT by a mitochondrial succinyl dehydrogenase in viable cells and has been shown to be a valuable method of addressing this purpose (Alley *et al.*, 1988; Scudiero *et al.*, 1988). Tumour cells (5000) in 100 μl standard growth medium were inoculated into each well of triplicate 96-microwell plates (Gibco), except for the first column, which served as blank. After 24 h RA and/or TNF-α were added to the desired final concentration resulting in the total amount of 200 μl medium per well. Column two served as control, containing tumour cells in culture medium (200 μl) without any drug supplement. After an incubation period of 120 h 0.25 mg of MTT dissolved in PBS (Serva, Germany) was added to each well and incubated for another 4 h. Formazan crystals were dissolved by exposure to DMSO for 10 min and colour intensity determined on a microculture plate reader (Titertek Multiscan Plus MK II) at 570 nm.

Values were expressed as:

% viable cells =

$$\frac{\text{Absorbance of test} - \text{absorbance of blank}}{\text{Absorbance of control} - \text{absorbance of blank}} \times 100$$

Data of MTT assay were analysed by means of *t*-tests.

*Receptor binding assay*

The binding assay for TNF receptors was essentially performed as described by Scheurich *et al.* (1987) with a small modification. Briefly, cells were harvested by exposure to 0.05% EDTA (Gibco) and washed three times. Triplicate samples of $2 \times 10^6$ cells each were incubated for 2 h at 4°C with various concentrations of [$^{125}$I]TNF-α (2–20 ng ml$^{-1}$) in a total volume of 0.3 ml of PBS (Gibco) containing 2% fetal calf serum and 0.02% sodium azide. In order to determine non-specific binding, radiolabelled TNF-α was mixed with a 200-fold excess of the unlabelled homologue before the addition of tumour cells. After incubation cells were washed three times, transferred into counting vials and counted by means of a gamma-counter (LKB-Beckmann, Germany). The number of TNF receptors per cell and the dissociation constant ($K_d$) were determined by Scatchard plot analysis.

*Determination of TNF mRNA and TNF receptor mRNA*

For Northern blot analysis aliquots of the epithelioid sarcoma cell lines were kept at −70°C until RNA preparation. Total cellular RNA was isolated by the guanidine–thiocyanate method as described by Chomczynski and Sacchi (1987). The RNA concentration was determined by photometry at 260 nm. The quality of total cellular RNA was verified in an ethidium bromide-stained agarose gel. Northern blot analysis was carried out with 25 μg of RNA of each sample under denaturing conditions with 1% formaldehyde–agarose gel. Before RNA transfer to nylon membranes the gel was stained with ethidium bromide and the equality of RNA amounts loaded in each lane was verified under UV light and photographed. In a second control step the complete RNA transfer from the gel to the nylon membrane was again verified under UV light. Afterwards, the RNA was hybridised with specific DNA probes. The DNA was labelled by incorporation of [$^{32}$P]dCTP using an oligolabelling kit (Pharmacia, Germany). The probes were obtained from the purified inserts of the following plasmids, which were kindly provided by Dr K Pfizenmaier, Germany: TNF-R1 (pAD-CMV1), insert:*Sal*I/*Xba*; TNF-R2 (pBluescript SK), insert: *Eco*RI; TNF-R2 (PCDM8), insert: *Xho*I/*Sca*I; TNF-α (pBR322), insert: *Eco*RI. Hybridisations were performed in 5 × SSC (1 × SSC = 0.15 M sodium chloride/0.015 M sodium citrate)/50% formamide/1 × Denhardt's solution (1 × Denhardt's solution = 0.02% bovine serum albumin/0.02% Ficoll/0.02% polyvinylpyrrolidone) and 100 μg denatured salmon sperm DNA per ml at 42°C for 18 h. Filters were washed in 2 × SSC/0.1% sodium dodecyl sulphate for 30 min at room temperature and in 0.1 × SSX/0.1% sodium dodecyl sulphate for 60 min at 60°C. Fluorography was carried out by exposure of Kodak X-Omat films for 10 days to dried filters at −70°C, in conjunction with intensifying screens. All experiments were done twice and the results could be reproduced.

*Determination of RA recptors*

RAR-α, RAR-β, and RAR-γ expression were determined by means of reverse transcriptase–PCR (RT–PCR). Total cellular RNA was prepared according to Stallcup and Washington (1983). PCR was run using RAR-specific primer sets as described by Ferrari *et al.* (1994) for 30 cycles (denaturation at 94°C for 1 min, primer annealing at 55°C for 1 min, extension at 72°C for 1 min). PCR products were transferred to a nylon membrane by Southern blotting. RAR-specific oligonucleotides (RAR-α: 5′-CCT TGC TTT GTC TGT CAG G-3′; RAR-β: 5′-TGC ATC CTC CAG GAG -AAA GCT-3′; RAR-γ: 5′-GGG TCA GCT CTT GT-G AAG GC-3′) were radiolabelled at the 5′-end with [γ-$^{32}$P]ATP using a commercially available T4 polynucleotide kinase (Pharmacia). Southern blots were prehybridised at 50°C for at least 3 h in 5 × SSPE, 2 × Denhardt's solution,

50 $\mu$g ml$^{-1}$ salmon sperm DNA, 50 $\mu$g ml$^{-1}$ *Escherichia coli* tRNA and 0.1% sodium dodecyl sulphate (SDS). After adding the radiolabelled oligonucleotide probes blots were incubated at 50°C for 18–22 h, followed by two washes in 2 × SSPE, 0.05% SDS, 5 × SSPE, and 0.1% SDS. Subsequently, autoradiography was performed. The calculated length of PCR products were 226 bp (RAR-$\alpha$); 388 bp (RAR-$\beta$); 351 bp (RAR-$\gamma$) respectively.

*Determination of TNF production*

For quantitative determination of TNF-$\alpha$ production by GRU-1A, GRU-1B and GRU-1C triplicate confluent tumour cell cultures were exposed to serum-free culture medium supplemented with 5 $\mu$g insulin ml$^{-1}$, 5 $\mu$g of transferrin and 5 pg of sodium selenite (Sigma). After 3 days, conditioned media were collected and concentrated by ultrafiltration using Centriprep-10 and Centricon-10 centrifugal microconcentrator devices (Amicon, Germany) according to the manufacturer's manual. The amount of TNF-$\alpha$ was determined on WEHI164cl13S cells in an 18 h assay in the presence of 1 $\mu$g ml$^{-1}$ actinomycin D as described by Vanhaesebroek *et al,* (1992). L929S, L929sAneg, L929sATmTNFWTC3, and L929M1.1high prod cells known to produce different amounts of TNF-$\alpha$ (Vanhaesebroeck *et al.*, 1991, 1992) were used in parallel as negative and positive controls respectively. The obtained results were calibrated with respect to both mouse and human TNF-$\alpha$ standards using a Curve Fit program.

### Results

In vitro *morphology*

Tumour cells of GRU-1A, GRU-1B, and GRU-1C exhibited a mainly epithelial-like, polygonal appearance and grew strictly anchorage-dependent as monolayers without evidence of cells piling up. Immunohistochemically, major differences could be detected in the quantitative expression of cytokeratin 18, vimentin and neurofilament proteins (Figure 1 and Table I). In order to investigate the effects of RA on tumour cell differentiation as defined by the distribution of these intermediate filaments, tumour cells were incubated for 5 days in medium supplemented with different concentrations of RA. As summarised in Table I, no significant change in the quantitative distribution of cytokeratin 18, vimentin and neurofilament proteins could be detected in GRU-1A, GRU-1B, or GRU-1C.

**Figure 1** Immunocytochemistry of GRU-1A (**a**, **d**, **g**), GRU-1B (**b**, **e**, **h**), and GRU-1C (**c**, **f**, **i**). Quantitative differences between the three clonal cell lines could be detected for cytokeratin 18 (**a**–**c**) and neurofilament proteins (**d**–**f**), while vimentin (**g**–**i**) disclosed a uniform reaction in all tumour cells of GRU-1A, GRU-1B and GRU-1C. The same expression pattern was observed after exposure to culture medium supplemented with different concentrations of RA. Scale bars, 25 $\mu$m.

*Plating efficiency*

After an incubation period of 14 and 28 days in standard growth medium or in medium supplemented with RA or TNF-α respectively, plating efficiency was determined for the clonal cell lines GRU-1A, GRU-1B and GRU-1C. As summarised in Table II, RA was able to reduce plating efficiency only in GRU-1B, whereas a similar reduction of plating efficiency was not observed in GRU-1A and GRU-1C. In contrast, TNF-α successfully reduced plating efficiency only in GRU-1A but not in GRU-1B. GRU-1C was affected by TNF-α only in the highest concentration and only after an incubation period of 28 days.

*Effects of RA and/or TNF-α on tumour cell proliferation (Figure 2)*

Proliferation analysis by MTT assay revealed growth-inhibitory effects for both RA and TNF -α in subpopulation GRU-1A. Thus, exposure to RA-containing medium resulted in a statistically significant dose-dependent growth inhibition to 82%±5% (0.1 μM RA, $P=0.03$) or 71%±5.5% (1 μM RA, $P=0.01$) of the control (=100%) after an incubation for 5 days *in vitro*. TNF-α proved to be even more effective than RA, inhibiting tumour cell proliferation dose dependently to 72%±6% (TNF-α 1 ng ml$^{-1}$, $P=0.02$), 57%±11% (TNF-α 10 ng ml$^{-1}$, $P=0.02$) and to 49%±4% (TNF-α 100 ng ml$^{-1}$, $P=0.002$) of the control (=100%) after exposure for 5 days. The effects of each singly applied compound could significantly be increased by combined exposure to both compounds. A maximal growth-inhibitory effect was achieved by the combination of RA (1 μM) and TNF-α (100 ng ml$^{-1}$), resulting in an inhibition of tumour cell proliferation to 40%±4% of the control ($P=0.001$), which was significantly more effective than the single application of RA ($P=0.006$) and TNF-α ($P=0.0002$) in equivalent concentrations.

GRU-1B cells proved to be sensitive only to RA, showing a growth-inhibitory effect to 91%±3% (RA 0.1 μM, $P=0.04$) and 81%±3% (1 μM RA, $P=0.006$) of the control (=100%). TNF-α, however, did not exhibit any effects on tumour cell proliferation of GRU-1B. The effects of RA on GRU-1B could not significantly be increased by a combined application of RA and TNF-α regardless of the concentrations used.

Unlike GRU-1A and GRU-1B, GRU-1C was resistant to any concentration of RA or TNF-α, applied either alone or in combination. Although differences to the control could be detected, the combination of RA (1 μM) and TNF-α (100 ng ml$^{-1}$) did not result in a significant antiproliferative effect owing to high standard deviations, which could not be reduced by five repeated assays.

*TNF-α receptor binding assay (Figure 2)*

Scatchard analysis revealed the highest number of TNF receptors in the GRU-1A cell line (1999 receptors per cell), while GRU-1B and GRU-1C exhibited 1063 receptors per cell and 1024 receptors per cell respectively. Furthermore, binding affinity could be shown to be essentially higher in GRU-1A ($K_d=3.9\times10^{-10}$, $r^2=0.98$) than in GRU-1B ($K_d=7.6\times10^{-10}$, $r^2=0.98$) and in GRU-1C ($K_d=8.5\times10^{-10}$, $r^2=0.91$).

*Expression of TNF-R1 and TNF-R2*

By Northern blot analysis all cell lines expressed TNF-R1 mRNA, whereas TNF-R2 mRNA was not detected at all (Figure 3a). In accordance with the results obtained by

**Table I** Quantitative distribution of cytokeratin 18 (CK18), neurofilament proteins (NR 4) and vimentin in GRU-1A, GRU-1B and GRU-1C

| | *CK 18 (%)* | *NR 4 (%)* | *Vimentin (%)* |
|---|---|---|---|
| GRU-1A | | | |
| Control | <1 | 100 | 100 |
| RA 0.1 μM | 0 | 100 | 100 |
| RA 1.0 μM | 0 | 100 | 100 |
| GRU-1B | | | |
| Control | <1 | 60 | 100 |
| RA 0.1 μM | 0 | 60 | 100 |
| RA 1.0 μM | <1 | 60 | 100 |
| GRU-1C | | | |
| Control | 40 | 80 | 100 |
| RA 0.1 μM | 45 | 70 | 100 |
| RA 1.0 μM | 20 | 60 | 100 |

The distribution pattern of these intermediate filaments did not significantly change by exposure to medium supplemented with different concentrations of retinoic acid.

**Table II** Plating efficiency of GRU-1A, GRU-1B and GRU-1C in standard growth medium or medium supplemented with various concentrations of RA or TNF-α respectively

| | *Plating efficiency (%)* | | | |
|---|---|---|---|---|
| | *14 days One cell/well* | *14 days Ten cells/well* | *28 days One cell/well* | *28 days Ten cells/well* |
| GRU-1A | | | | |
| Control | 5 | 38 | 9 | 77 |
| RA 0.1 μM | 8 | 31 | 27 | 89 |
| RA 1.0 μM | 3 | 23 | 8 | 75 |
| TNF-α 1 ng ml$^{-1}$ | 2 | 22 | 3 | 65 |
| TNF-α 10 ng ml$^{-1}$ | 1 | 6 | 3 | 23 |
| TNF-α 100 ng ml$^{-1}$ | 0 | 6 | 2 | 8 |
| GRU-1B | | | | |
| Control | 7 | 11 | 14 | 74 |
| RA 0.1 μM | 0 | 4 | 2 | 48 |
| RA 1.0 μM | 0 | 2 | 4 | 38 |
| TNF-α 1 ng ml$^{-1}$ | 4 | 20 | 10 | 75 |
| TNF-α 10 ng ml$^{-1}$ | 3 | 18 | 5 | 60 |
| TNF-α 100 ng ml$^{-1}$ | 8 | 18 | 13 | 55 |
| GRU-1C | | | | |
| Control | 8 | 32 | 19 | 95 |
| RA 0.1 μM | 6 | 29 | 20 | 94 |
| RA 1.0 μM | 8 | 50 | 17 | 92 |
| TNF-α 1 ng ml$^{-1}$ | 16 | 33 | 28 | 82 |
| TNF-α 10 ng ml$^{-1}$ | 12 | 39 | 16 | 89 |
| TNF-α 100 ng ml$^{-1}$ | 5 | 34 | 7 | 53 |

Scatchard analysis the strongest signal for TNF-R1 mRNA was found in GRU-1A, whereas GRU-1B and GRU-1C expressed TNF-R1 mRNA to a significantly lower extent, not differing between these two cell lines. Since co-incubation of GRU-1A cells in TNF-α and RA resulted in a significantly stronger antiproliferative effect when compared with each single compound, the effect of RA on the expression of TNF-R1 mRNA was investigated, showing that an incubation period of 5 days in 1 μM RA did not significantly influence TNF-R1 mRNA expression.

### *TNF production*

Conditioned media from GRU-1A, GRU-1B and GRU-1C did not contain cytotoxic activity towards WEHI164c113S cells, whereas conditioned media of L929sATmTNFWTC3 and L929M1.1high prod cells used as positive controls exhibited marked cytotoxic activities in the range of $3.6\times10^4-4.4\times10^6$ IU $ml^{-1}$. Furthermore, no TNF-α mRNA could be detected in GRU-1A, GRU-1B, or GRU-1C as evidenced by Northern blot analysis (data not shown).

**Figure 2** MTT proliferation assays (left) and Scatchard plot analysis (right) for GRU-1A, GRU-1B and GRU-1C. The number of tumour cells is presented as proportion of the control (=100%). $K_d$, dissociation constant; c.p.m., counts $min^{-1}$; TB, total binding; SB, specific binding; NSB, non-specific binding. Bound and free refer to concentrations of bound and free TNF.

**Figure 3** (**a**) Expression of TNF-R1 and TNF-R2 mRNA in GRU-1A (1, after incubation in 1 $\mu$M RA for 5 days; 2, non-treated GRU-1A cells), GRU-1B (3) and GRU-1C (4) as detected by Northern blot analysis. (*Ethidium bromide-stained gel showing equal amounts of RNA in each lane.) (**b**) Expression of RAR-$\alpha$, RAR-$\beta$, and RAR-$\gamma$ in GRU-1A (1), GRU-1B (2) and GRU-1C (3) as determined by RT–PCR and subsequent receptor-specific oligonucleotide hybridisation.

*Expression of RA receptors*

By RT–PCR and subsequent oligonucleotide hybridisation (Figure 3b) RAR-$\alpha$ could be detected exclusively in GRU-1B whereas RAR-$\beta$ was not expressed in any cell line. RAR-$\gamma$ mRNA could be found in GRU-1A and GRU-1C but not in GRU-1B.

### Discussion

Since the first description of epithelioid sarcoma as a unique entity by Enzinger in 1970, little progress has been achieved in the therapy of this malignancy. At present, radical excision or amputation is still regarded as the most effective way of initial treatment, although there is a 63–77% rate of recurrence after initial surgical procedure and a 45–58% incidence of metastasis (Prat *et al.*, 1978; Chase and Enzinger, 1985). Irradiation and conventional chemotherapy with agents such as doxorubicin, vincristine, cytoxan, actinomycin D and methotrexate as well as platinol, oncovin and interferon have proved to be ineffective (Chase and Enzinger, 1985).

The present study clearly shows that both RA and TNF-$\alpha$ are able to exhibit significant antiproliferative effects on epithelioid sarcoma *in vitro*, although the results differ markedly between the three clonal subpopulations. Thus, RA dose-dependently inhibited tumour cell proliferation of GRU-1A and GRU-1B after an incubation period of 5 days, whereas no effect was seen in GRU-1C. The effects of RA are known to be mediated through two classes of nuclear retinoid receptors: retinoic acid receptors (RARs) which bind all-*trans* retinoic acid (RA), the compound used in our study; and retinoid X receptors (RXRs), which bind 9-*cis* retinoic acid with high affinity (Chambon, 1993; Mangelsdorf, 1994). Both classes of receptors have been found to consist of at least three different isoforms each ($\alpha$, $\beta$, $\gamma$), the functional impact of these isoforms still far from being elucidated (Chambon, 1993; Mangelsdorf, 1994). In our tumour model RAR-$\alpha$ was expressed only in GRU-1B, whereas RAR-$\beta$ was not detected in any cell line. RAR-$\gamma$ was found in GRU-1A and GRU-1C. No direct correlation, however, between the RA-induced growth inhibition, observed in GRU-1A and GRU-1B, and the expression pattern of RARs became evident. Similar results have been reported by van der Leede *et al.* (1993) for other tumour models. Interestingly in this context, a marked reduction of plating efficiency by RA was found exclusively in GRU-1B, the only cell line expressing RAR-$\alpha$, while GRU-1A and GRU-1C proved to be resistant. Nevertheless, it remains to be determined whether the association between RAR-$\alpha$ expression and reduction of plating efficiency is causative rather than coincidental.

In other tumour models growth-inhibitory effects of RA have previously been shown to be coupled with a simultaneous induction of differentiation (Mummery *et al.*, 1984; Gabbert *et al.*, 1988; Joyce and Steer, 1992). In spite of significant growth-inhibitory effects, however, RA failed to exhibit any differentiation-inductive effects in GRU-1A and GRU-1B. Thus, immunohistochemical analysis revealed no change in the distribution pattern of cytokeratin 18, vimentin or neurofilament proteins, indicative for epithelial, mesenchymal and neural differentiation respectively. The uncoupling of growth inhibition and differentiation induction observed in GRU-1A and GRU-1B after exposure to RA could result from the complexity of RA signal transduction pathways. Thus, receptor heterodimerisation, which has recently been reported to occur between RARs and RXRs (Marks *et al.*, 1992; Nagpal *et al.*, 1993), may result in multiple mutually distinct receptors, each of which presumably activates specific target genes in the regulation of proliferation and differentiation.

TNF-$\alpha$ exhibited a highly significant antiproliferative effect in GRU-1A, whereas GRU-1B and GRU-1C proved to be TNF-resistant. TNF-$\alpha$ is known to exhibit a multitude of different effects upon binding to two distinct high-affinity receptors, TNF-R1 and TNF-R2 (Tartaglia and Goeddel, 1992; Sidhu and Bollon, 1993), the specific functional impact of each receptor type on growth regulation and cytotoxicity still being under discussion (Heller *et al.*, 1992; Tartaglia *et al.*, 1993; Grell *et al.*, 1993; Higuchi and Aggarwal, 1994). In our tumour model, however, the antiproliferative effect of TNF-$\alpha$ was exclusively mediated by TNF-R1 since no TNF-R2 mRNA was detectable. Furthermore, a positive correlation between TNF-$\alpha$-induced growth inhibition and both the number of TNF-R1 and receptor affinity became evident. In addition, it was excluded on both the mRNA and protein level that TNF resistance in GRU-1B and GRU-1C was due to endogenous TNF-$\alpha$ production, as has been reported from other tumour models (Vanhaesebroeck *et al.*, 1992), since all subpopulations of GRU-1 proved to be TNF negative. Finally, it was shown that the combined exposure to RA

and TNF-α resulted in an additive rather than a synergistic antiproliferative effect in GRU-1A. In accordance with this observation, RA did not significantly alter TNF-R1 mRNA expression in the same cell line, suggesting that the antiproliferative effects of both compounds might be mediated by independent signal transduction pathways.

In conclusion, RA and TNF-α have been shown to exhibit significant and in part additive growth-inhibitory effects in human epithelioid sarcoma, the response, however, markedly differing between the three clonal subpopulations of our tumor model. The antiproliferative effects of TNF-α were mediated by TNF-R1 and correlated with both the number of TNF-R1 and receptor affinity. Therefore, the clonal subpopulations GRU-1A, GRU-1B and GRU-1C provide an excellent human *in vitro* model to investigate TNF resistance and TNF signalling pathways as well as the susceptibility of epithelioid sarcoma for new therapeutic strategies, taking into account tumour heterogeneity.

**Acknowledgements**
We would like to express our appreciation to U Herian, M van den Hemel, P Pulkowski, M Ringler and F Rinschedde for their excellent technical assistance. We are grateful to Professor Dr G Hommel for his statistical evaluations. F van Roy is Research Director with the Belgian FNRS.

### References

ALLEY MC, SCUDIERO DA, MONKS A, CZERWINSKI MJ, FINE DL, ABBOTT BJ, MAYO JG, SHOEMAKER RH AND BOYD MR. (1988). Feasibility of drug screening with panels of human tumor cell lines using a microculture tetrazolium assay. *Cancer Res.*, **48,** 589-601.

BEYAERT R AND FIERS W. (1994). Molecular mechanisms of tumor necrosis factor-induced cytotoxicity – what we do understand and what we do not. *FEBS Lett.*, **340,** 9-16.

CHAMBON P. (1993). The molecular and genetic dissection of the retinoid signalling pathway. *Gene*, **135,** 223-228.

CHASE DR AND ENZINGER FM. (1985). Epithelioid sarcoma; diagnosis, prognostic indicators, and treatment. *Am. J. Surg. Pathol.*, **9,** 241-263.

CHROMCZYNSKI P AND SACCHI N. (1987). Single-step method of RNA isolation by acid guanidinium thiocyanate-phenol-chloroform extraction. *Anal. Biochem.*, **162,** 156-159.

DEBUS E, WEBER K AND OSBORN M. (1983). Monoclonal antibodies specific for glial fibrillary acidic (GFA) protein and for each of the neurofilament triplet polypeptides. *Differentiation*, **25,** 193-203.

ENGERS R, GERHARZ CD, MOLL R, POHL A, SARBIA M AND GABBERT HE. (1994). Interclonal heterogeneity in a human epithelioid sarcoma cell line (GRU-1). *Int. J. Cancer*, **59,** 548-553.

ENZINGER FM. (1970). Epithelioid sarcoma. A sarcoma simulating a granuloma or a carcinoma. *Cancer*, **26,** 1029-1041.

FERRARI N, PFEFFER U, TOSETTI F, BRIGATI C AND VIDALI G. (1994). An improved RT-PCR protocol for the quantification of human retinoic acid receptor RNA. *Exp. Cell. Res.*, **211,** 121-126.

GABBERT HE, GERHARZ CD, BIESALSKI HK, ENGERS R AND LULEY C. (1988). Terminal differentiation and growth inhibition of a rat rhabdomyosarcoma cell line (BA-HAN-1C) in vitro after exposure to retinoic acid. *Cancer Res.*, **48,** 5264-5269.

GERHARZ CD, MOLL R, RAMP U, MELLIN W AND GABBERT HE. (1990). Multidirectional differentiation in a newly established human epithelioid sarcoma cell line (GRU-1) with co-expression of vimentin, cytokeratins and neurofilament proteins. *Int. J. Cancer*, **45,** 143-152.

GERHARZ CD, BRACKE ME, MAREEL MM AND GABBERT HE. (1993). Modulation of invasive potential in different clonal subpopulations of a rat rhabdomyosarcoma cell line (BA-HAN-1) by differentiation induction. *Clin. Exp. Metastasis*, **11,** 55-67.

GRELL M, SCHEURICH P, MEAGER A AND PFIZENMAIER K. (1993). TR60 and TR80 tumor necrosis factor (TNF)-receptors can independently mediate cytolysis. *Lymph. Cytok. Res.*, **12,** 143-148.

HALEVY O AND LERMAN O. (1993). Retinoic acid induces adult muscle cell differentiation mediated by retinoic acid receptor-α. *J. Cell Physiol.*, **154,** 566-572.

HELLER RA, SONG K, FAN N AND CHANG DJ. (1992). The p70 tumor necrosis factor receptor mediates cytotoxicity. *Cell*, **70,** 47-56.

HIGUCHI M AND AGGARWAL BB. (1994). Differential roles of two types of the TNF receptor in TNF-induced cytotoxicity, DNA fragmentation, and differentiation. *J. Immunol.*, **152,** 4017-4025.

JOYCE DA AND STEER JH. (1992). Differentiation of the U-937 promonocytic cell line induced by phorbol myristate acetate or retinoic acid: effect of aurothiomolate. *Agents Actions*, **37,** 305-310.

LEJEUNE F, LIENARD D, EGGERMONT A, SCHRAFFORDT-KOOPS H, KROON B, GERAIN J, ROSENKAIMER F AND SCHMITZ P. (1994*a*). Clinical experience with high-dose tumor necrosis factor alpha in regional therapy of advanced melanoma. *Circ. Shock*, **43,** 191-197.

LEJEUNE F, LIENARD D, EGGERMONT A, SCHRAFFORDT-KOOPS H, ROSENKAIMER F, GERAIN J, KLAASE J, KROON B, VANDERVEKEN J AND SCHMITZ P. (1994*b*). Rationale for using TNF alpha and chemotherapy in regional therapy of melanoma. *J. Cell. Biochem.*, **56,** 52-61.

MCGARVEY TW, SILBERMAN S AND PERSKY B. (1990). The effect of butyric acid and retinoic acid on invasion and experimental metastasis of murine melanoma cells. *Clin. Exp. Metastasis*, **8,** 433-448.

MANGELSDORF DJ. (1994). Vitamin A receptors. *Nutr. Rev.*, **52,** 32-44.

MARKS SM, HALLENBECK PL, NAGATA T, SEGARS JH, APPELLA E, NIKODEM VM AND OZATO K. (1992). H-2RIIBP (RXRβ) heterodimerization provides a mechanism for combinatorial diversity in the regulation of retinoic acid and thyroid hormone responsive genes. *EMBO J.*, **11,** 1419-1435.

MOLL R, ACHTSTÄTTER T, BECHT E, BALCAROVA-STÄNDER J, ITTENSOHN M AND FRANKE WW. (1988). Cytokeratins in normal and malignant transitional epithelium: maintenance of expression of urothelial differentiation features in transitional-cell carcinomas and bladder carcinoma cell lines. *Am. J. Pathol.*, **132,** 123-144.

MUMMERY CL, VAN DEN BRINK CE, VAN DER SAAG PT AND DE LAAT SW. (1984). The cell cycle, cell death, and cell morphology during retinoic-acid induced differentiation of embryonal carnicoma cells. *Dev. Biol.*, **104,** 297-307.

NAGPAL S, FRIANT S, NAKSHATRI H AND CHAMBON P. (1993). RARs and RXRs: evidence for two autonomous transactivation functions (AF-1 and AF-2) and heterodimerization in vivo. *EMBO J.*, **12,** 2349-2360.

PITZ S, MOLL R, STÖRKEL S AND THOENES W. (1987). Expression of intermediate filament proteins in subtypes of renal cell carcinomas and in renal oncocytomas. *Lab. Invest.*, **6,** 642-653.

PRAT J, WOODRUFF JM AND MARCOVE RC. (1978). Epithelioid sarcoma: an analysis of 22 cases indicating the prognostic significance of vascular invasion and regional lymph-node metastasis. *Cancer*, **41,** 1472-1487.

PUSZTAI L, LEWIS CE AND MCGEE JO. (1993). Growth arrest of the breast cancer cell line, T47D, by TNFalpha – cell cycle specificity and signal transduction. *Br. J. Cancer*, **67,** 290-296.

REEVES BR, FISHER C, SMITH S, COURTENAY VD AND ROBERTSON D. (1987). Ultrastructural, immunocytochemical, and cytogenetic characterization of a human epithelioid sarcoma cell line (RM-HS1). *J. Natl Cancer Inst.*, **78,** 7-18.

RUTKA JT, GIBLIN JR, BERENS ME, BAR-SHIVA E, TOKUDA K, MCCULLOCH JR, ROSENBLUM ML, EESSALU TE, AGGARWAL BB AND BODELL WJ. (1988). The effects of human recombinant tumor necrosis factor on glioma-derived cell lines: cellular proliferation, cytotoxicity, morphological and radioreceptor studies. *Int. J. Cancer*, **41,** 573-582.

SCHEURICH P, THOMA B, ÜCER U AND PFIZENMAIER K. (1987). Immunoregulatory activity of recombinant human tumor necrosis factor (TNF)-α: induction of the TNF receptors on human T cells and TNF-α-mediated enhancement of T cell response. *J. Immunol.*, **138,** 1786-1790.

SCUDIERO DA, SHOEMAKER RH, PAULL KD, MONKS A, TIERNEY S, NOFZINGER TH, CURRENS MJ, SENIFF D AND BOYD MR. (1988). Evaluation of a soluble tetrazolium/formazan assay for cell growth and drug sensitivity in culture using human and other tumor cell lines. *Cancer Res.*, **48,** 4827-4833.

SIDHU RS AND BOLLON AP. (1993). Tumor necrosis factor activities and cancer therapy – a perspective. *Pharmacol. Ther.*, **57,** 79-128.

SONOBE H, FURIHATA M, IWATA J, OKA T, OHTSUKI Y, HAMASATO S AND FUJIMOTO S. (1993). Morphological characterization of a new human epithelioid sarcoma cell line, ES020488, in vitro and in vivo. *Virchows Arch. B.*, **63**, 219–225.

STALLCUP MR AND WASHINGTON LD. (1983). Region-specific initiation of mouse mammary tumor virus RNA synthesis by endogenous RNA polymerase II in preparations of cell nuclei. *J. Biol. Chem.*, **258**, 2802–2807.

SUGARMAN BJ, AGGARWAL BB, HASS PE, FIGARI IS, PALLADINO JR MA AND SHEPARD HM. (1985). Recombinant human tumor necrosis factor-α: effects on proliferation of normal and transformed cells in vitro. *Science*, **230**, 943–945.

TARTAGLIA LA AND GOEDDEL DV. (1992). Two TNF receptors. *Immunol. Today*, **13**, 151–153.

TARTAGLIA LA, ROTHE M, HU Y-F AND GOEDDEL DV. (1993). Tumor necrosis factor's cytotoxic activity is signaled by p55 TNF receptor. *Cell*, **73**, 213–216.

VAN DER LEEDE BM, VAN DEN BRINK CE AND VAN DER SAAG PT. (1993). Retinoic acid and retinoid X receptor expression in retinoic acid-resistant human tumor cell lines. *Mol. Carcinogen.*, **8**, 112–122.

VANHAESEBROECK B, VAN BLADEL S, LENAERTS A, SUFFYS P, BEYAERT R, LUCAS R, VAN ROY F AND FIERS W. (1991). Two discrete types of tumor necrosis factor-resistant cells derived from the same cell line. *Cancer Res.*, **51**, 2469–2477.

VANHAESEBROECK B, DECOSTER E, VAN OSTADE X, VAN BLADEL S, LENAERTS A, VAN ROY F AND FIERS W. (1992). Expression of an exogenous tumor necrosis factor (TNF) gene in TNF-sensitive cell lines confers resistance to TNF-mediated cell lysis. *J. Immunol.*, **148**, 2785–2794.